# Prognostic Impact of Membranous/Nuclear Epidermal Growth Factor Receptor Localization in Clear Cell Renal Cell Carcinoma

**DOI:** 10.3390/ijms22168747

**Published:** 2021-08-14

**Authors:** Maria Rosaria Muroni, Silvia Ribback, Giovanni Sotgiu, Nils Kroeger, Laura Saderi, Andrea Angius, Paolo Cossu-Rocca, Maria Rosaria De Miglio

**Affiliations:** 1 Department of Medical, Surgical and Experimental Sciences, University of Sassari, Via P. Manzella, 4, 07100 Sassari, Italy; mrmuroni@uniss.it (M.R.M.); gsotgiu@uniss.it (G.S.); lsaderi@uniss.it (L.S.); rocco@uniss.it (P.C.-R.); 2 Institut fuer Pathologie, Universitaetsmedizin Greifswald, Friedrich-Loeffler-Str. 23e, 17475 Greifswald, Germany; silvia.ribback@uni-greifswald.de; 3 Klinik und Poliklinik fuer Urologie, Universitaetsmedizin Greifswald, Sauerbruchstr, 17475 Greifswald, Germany; kroegern@uni-greifswald.de; 4 Istituto di Ricerca Genetica e Biomedica (IRGB), CNR, Cittadella Universitaria di Cagliari, 09042 Monserrato, Italy; 5 Surgical Pathology Unit, Department of Diagnostic Services, “Giovanni Paolo II” Hospital, ASSL Olbia-ATS Sardegna, 07026 Olbia, Italy

**Keywords:** clear cell renal cell carcinoma, membranous-cytoplasmic EGFR, nuclear EGFR, SGLT1, prognosis, survival

## Abstract

EGFR is overexpressed in the majority of clear cell renal cell carcinomas (CCRCCs). Although EGFR deregulation was found to be of great significance in CCRCC biology, the EGFR overexpression is not associated with EGFR-targeted therapy responsiveness. Moreover, the prognostic role of EGFR expression remains controversial. In the present study, we evaluated the role played by EGFR overexpression in CCRCC and its prognostic significance associated with different immunohistochemical localization patterns. In our study, the Total Score (TS) related to membranous-cytoplasmic EGFR expression showed a significant correlation with grade, pathologic stage (pT), and Stage, Size, Grade, and Necrosis (SSIGN) score, and a negative correlation with nuclear EGFR expression. No significant correlations were shown between nuclear EGFR and clinic-pathological features. Additionally, a correlation between SGLT1 expression levels and pT was described. Multivariate analysis identifies pT and SSIGN score as independent prognostic factors for CCRCC. A significantly increased survival rate was found in the case of positive expression of nuclear EGFR and SGLT1. Based on our findings, SGLT1 and nuclear EGFR overexpression defines a subgroup of CCRCC patients with good prognosis. Membranous-cytoplasmic EGFR expression was shown to be a poor prognostic factor and could define a CCRCC subgroup with poor prognosis that should be responsive to anti-EGFR therapies.

## 1. Introduction

Clear cell renal cell carcinoma (CCRCC) is the most aggressive renal cell carcinoma (RCC), representing ~85% of all RCC types. Synchronous metastases are likely to be found in 20–30% of cases. The 20% of patients that undergo nephrectomy may develop metastasis or recurrence during the follow-up [1]. Despite strong advances in therapeutics, survival rates remain poor for metastatic CCRCC due to resistance to chemo- and radio-therapy, including targeted therapies [2]. Recently, the introduction of immune checkpoint inhibitors (ICIs, i.e., T-cell checkpoint blockage with PD-1/PDL-1 or CTLA-4 antibodies) as single-agent or in combination with other ICIs, or with recent generation of VEGF tyrosine kinase inhibitors (TKIs), has shown impressive survival benefits in metastatic RCC [3]. The survival benefit provided by first line ICI-TKI combinations vs. sunitinib monotherapy has been proved in all metastatic RCC patients regardless of clinico-pathological data [4].

Although the Fuhrman nuclear grade is a reliable prognostic factor, defining a precise individual prognosis is still challenging; the establishment of molecular mechanisms might help in the clinical decision-making process.

EGFR immunoreactivity can be found in 50% to 90% of CCRCC [5,6,7,8,9,10,11]. EGFR overexpression is associated with high grades, stages and Stage, Size, Grade, and Necrosis (SSIGN) score [7,10,11,12,13]. High levels of EGFR expression is not associated with EGFR-targeted therapy responsiveness [14,15,16]. Increasing evidence exhibits the relationship between EGFR-targeted therapy response and peculiar genetic abnormalities, such as gene mutations or gene amplification in various human tumors [17,18]. We demonstrated the absence of mutations in exons 18 to 24 and/or presence of EGFR-variant III (EGFRvIII), or gene amplification in all CCRCCs analyzed. EGFR overexpression was present in 38.2% of tumors [11]. We proved activation of the EGFR kinase-independent function related to SGLT1 overexpression, which interacts with EGFR as a target increasing cancer cell metabolism and neoplastic progression [11].

EGFR can be found in the plasma membrane, cytoplasm and nucleus. Nuclear-EGFR acts as a transcription factor regulating gene expression and cellular processes involved in tumor biology [19,20,21,22,23]. A relationship between nuclear EGFR and breast, oropharyngeal, ovarian, bladder cervical, and renal cancers has been highlighted [24,25,26,27,28,29]. Membranous and cytoplasmic EGFR localization was related to different prognostic patterns [10,30,31].

The aim of the present study was to assess the role of EGFR overexpression in CCRCC and its prognostic significance in association with different immunohistochemical patterns.

## 2. Results

One hundred and twenty patients with CCRCC were retrospectively recruited (Table 1). The mean (SD) age was 62 (±10.9) years and 85.8% were diagnosed when aged > 50 years, with a higher prevalence of males (59.2%). The median tumor size was 6 cm, with 56.7% of tumors showing a size > 5 cm. Necrosis was present in 44.0% of the cases. Overall, 48.3% of CCRCCs were classified as T1, 14.2% as T2, and 37.5% as T3. Additionally, 37.5% were classified as pN0, 4.2% as pN1, and 2.5% as pN2. The percentage of patients that presented distant metastasis was 27.5%. Moreover, 16.1% of tumors were stage I, 7.4% stage II, 34.6% stage III, and 42.0% stage IV, while 10.8% of CCRCCs were G1, 46.7% G2, 35.0% G3 and 7.5% G4.

Finally, 83.3% showed a SSIGN score of 0–9. Five-year follow-up data showed no evidence of disease (NED) in 79 (66.4%) patients and progression in 40 patients. Of patients with progression, 32 (26.9%) developed metastases and were still alive with disease (AWD) and 8 (6.7%) with distant metastases were disease-related deaths (DOD).

### 2.1. Immunohistochemical Analysis

Membranous-cytoplasmic EGFR expression was present in 92.5% of cases, with staining intensity ranging from 1+ to 3+. The percentage of positive cells varied from 20% to 100%. The Total Score EGFR ranged between 0 and 300, with a mean (SD) score of 177.5 (78.1).

Nuclear EGFR expression was found in 19.3% of the tumors, with staining intensity ranging from 1+ to 3+, and the percentage of positive cells ranged from 20% to 80%.

**Figure 1:**
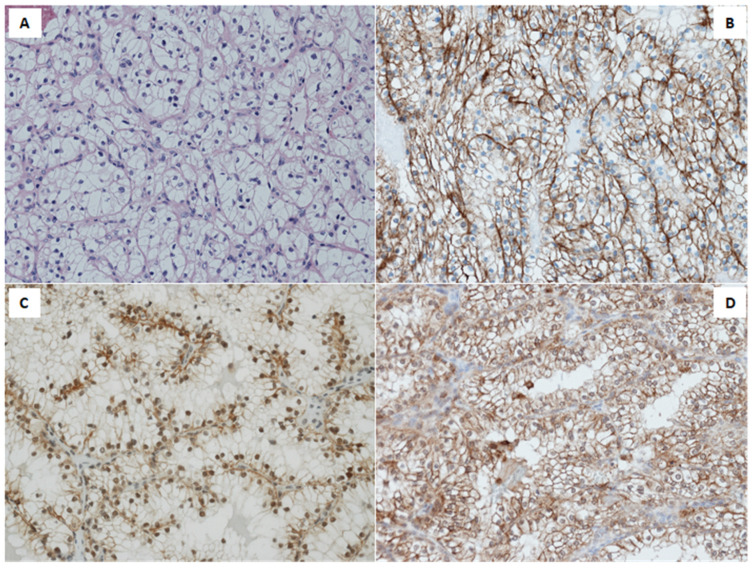
Morphologic and immunohistochemical features of Clear Cell Renal Cell Carcinoma. (**A**) Hematoxylin and Eosin stain shows typical CCRCC morphologic features; (**B**) Immunohistochemistry for EGFR (clone 2-18C9) displaying diffuse and intense membranous immunoreactivity; (**C**) Immunohistochemistry for EGFR (polyclonal sc-03) displaying intense nuclear immunoreactivity; (**D**) Immunohistochemistry for SGLT1 on CCRCC showing diffuse and intense, predominantly membranous immunoreactivity.

Immunohistochemistry (IHC) revealed a membranous EGFR expression predominantly in high nuclear grade, poorly differentiated tumors. Conversely, nuclear EGFR was stated in low nuclear grade, well differentiated tumors, as shown in Figure 1B,C.

SGLT1 expression was present in 85.0% of the tumors, with staining intensity ranging from 1+ to 3+. The percentage of positive cells ranged from 15% to 90% (Figure 1D). Co-expression of membranous-cytoplasmic EGFR and SGLT1 accounts for 77.5% of the cohort.

### 2.2. Statistical Analysis

Total score related to membranous-cytoplasmic EGFR expression levels showed a significant correlation with grade (r = 0.35; *p* = 0.0001), pT (r = 0.21; *p* = 0.020), and SSIGN score (r = 0.25; *p* = 0.007) (see Table 2), while a negative correlation was present between TS-EGFR and nuclear EGFR expression.

No significant association between nuclear EGFR and clinic-pathological features (Table 3) was shown. We found a correlation between SGLT1 expression levels and pT (Table 4).

Multivariate analysis identified pT and SSIGN score as independent prognostic factors for CCRCC (Table 5).

No statistically significant differences were detected when different combinations of membranous-cytoplasmic EGFR/nuclear EGFR, membranous-cytoplasmic EGFR/SGLT1 and nuclear EGFR/SGLT1 expression levels were observed in CCRCC patients with NED compared to patients with progression of disease at 5 years follow-up (Table 6). However, CCRCC patients with nuclear EGFR/SGLT1 double negative experienced a poor prognosis.

**Figure 2:**
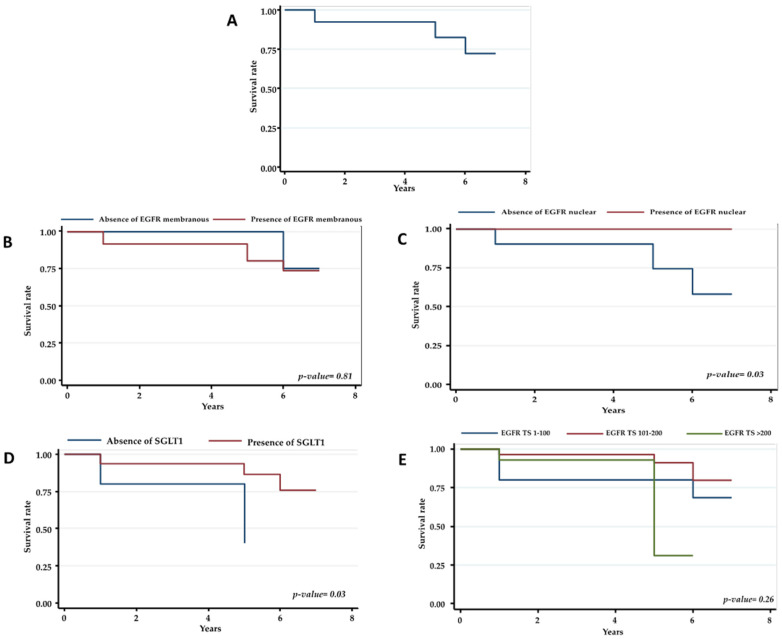
Kaplan–Meier curves for overall survival. (**A**) distribution of overall survival (**B**) overall survival according to absence/presence of immunostaining for membranous-cytoplasmic EGFR (**C**) overall survival according to three TS-EGFR groups, (**D**) overall survival according to absence/presence of immunostaining for nuclear EGFR, (**E**) overall survival according to absence/presence of immunostaining for SGLT1.

A significantly increased survival was found on positive expression of nuclear EGFR (*p*: 0.03) and SGLT1 (*p*: 0.03) (Figure 2).

## 3. Discussion

Fuhrman nuclear grade and pathological stage are adopted in the clinical management of CCRCC, although they are currently unable to properly predict the disease outcome in patients and the biologic tumor aggressiveness. Our study demonstrates that EGFR expression is an important prognostic factor. Membranous-cytoplasmic EGFR overexpression accounts for 92.5% of the cohort. TS associated with membranous-cytoplasmic EGFR expression levels showed significant correlations with unfavorable clinico-pathological parameters (i.e., grade, pT, and SSIGN score).

These results are in keeping with recent IHC studies correlating EGFR overexpression and unfavorable clinico-pathological features of CCRCC, such as tumor size and SSIGN score [11], high tumor grade and stage, poorly differentiated tumors, poor prognosis [10,29,31,32,33], invasion [30], large tumor size and shorter survival [33]. Despite the fact that anti-EGFR therapy does not appear to be effective in the absence of EGFR-related genetic anomalies [16,34,35,36], our previous findings demonstrated the activating role of EGFR overexpression on downstream signaling pathways and its kinase-dependent function [11].

Our data show overexpression of SGLT1 together with co-expression of EGFR in 77.5% of tumors, suggesting that EGFR kinase-independent function might contribute to tumor progression. Weiuha et al. revealed that EGFR sustains the basal intracellular glucose level preventing autophagic neoplastic cells death [37]. A growing body of evidence on integrated molecular omics profiling supports the definition of CCRCC as a metabolic disease. Metabolic reprogramming involving the glucose metabolism has been identified in CCRCC. Increased glycolysis and partition of glycolytic flux, increased pentose phosphate pathway (PPP), and decreased TCA cycle are responsible for tumor promotion through the rerouting of sugar metabolism [38,39].

In support of the involvement of bioenergetic alterations in CCRCC biology, overexpression of NDUFA4L2 in CCRCC blocks oxidative phosphorylation, reduces ROS production, and increases cellular antioxidants levels promoting progression and drug resistance [40]. Recently, Lucarelli et al. identified a lipid metabolism reprogramming associated with a switch in adipogenic gene signatures in CCRCC, with accumulation of very long-chain FAs and PUFAs, sustained by overexpression of SCD1 and ELOVLs [41].

Consequently, the EGFR-independent kinase function could give tumor cells enhanced survival and growth capacity by contributing to metabolism deregulation, even in the presence of chemotherapeutic agents and TKIs.

The correlation between SGLT1 immunohistochemical intensity levels and CCRCC with pT1 might suggest a prognostic role of SGLT1. Based on the latest findings, the increased activity of the EGFR-SGLT1 interaction could be responsible for glucose flux in neoplastic cells before reprogramming their metabolism by overlapping genetic and epigenetic aberrations. A total of 19.3% of tumors show nuclear EGFR overexpression with no correlation with clinico-pathological features. Nuclear EGFR expression and low nuclear Fuhrman grade together with well differentiated tumors revealed a good prognosis. EGFR kinase-function, through activation of multiple tyrosine kinase signaling pathways, is involved in the proliferation of poorly differentiated cells while nuclear EGFR expression is reduced in the cells of high nuclear grade [29].

Downregulation of nuclear EGFR could promote progression by inducing loss of interaction with the DNA-dependent protein kinase leading to the repair of a DNA double strand break [42,43]. The primary function of EGFR in organogenesis and physiology of kidneys and our results support the hypothesis of Ahel et al. [29]: intracellular trafficking and regulation of EGFR protein could be altered in CCRCC, and nuclear EGFR signaling is probably involved in controlled proliferation in low nuclear grade of well differentiated tumors.

Our results showed that SSIGN score and tumor size are independent prognostic factors for CCRCC.

We described for the first time a positive correlation between overexpression of nuclear EGFR and SGLT1 and good survival outcome, compared with patients showing negative expression of nuclear EGFR and SGLT1. Our results are supported by studies in solid tumors showing a negative correlation between nuclear EGFR expression and survival in breast ovarian, uterine cervix, bladder, and oropharyngeal squamous cell cancer [24,25,26,27,28].

This study needs to consider some limitations, mainly inherent to its retrospective design. Thus, we could not retrieve information on vital status at follow-up because it was not originally included in the medical records. That missing information may affect the statistical power of the associations evaluated. Moreover, the analysis should be extended to more patients in the coming years to strengthen and replicate our results.

The findings of our study highlight the complex role performed by EGFR in the pathogenesis of CCRCC. Considering the retrospectivity of the study, in vitro experiments will be organized to analyze the effective mechanisms of EGFR-SGLT1 interaction, especially in neoplastic cells with different level of differentiation. It will be important to determine the exact molecular role of nuclear EGFR in CCRCC. Knowledge of the molecular mechanisms should allow the development of new therapeutic strategies in CCRCC alone or with known drugs. Moreover, a clinical utility of these data as prognostic biomarkers should be evaluated in the near future in in vivo studies.

## 4. Materials and Methods

### 4.1. Recruitment of CCRCC Patients

The study was conducted according to ethics criteria of the World Medical Association (Declaration of Helsinki). According to the Italian guidelines for observational studies (G.U. n. 76. 31-3-2008), ethical approval and informed consent are not required for this study. All samples were anonymized.

Tumors selected from the Histopathology Departments archives of Cagliari Hospital, Italy and from the Departments of Urology in Greifswald and Teubingen, Germany and Austria, were evaluated by experienced pathologists following the currently available classification and staging systems [44]. Four µm thick tissue sections of formalin-fixed, paraffin embedded (FFPE) specimens were used for haematoxylin and eosin stains and IHC analysis. Data on age, sex, tumor size, TNM classification, Fuhrman nuclear grade, stage necrosis, SSIGN score, recurrence, metastasis and death were collected and included in a database.

### 4.2. Immunohistochemistry

FFPE serial tumor sections in 1–2 µm thickness were stained in automated immunostainer for SGLT1 (1:100 overnight incubation; Rabbit Polyclonal, Novus Biological, Littleton, CO, USA) and EGFR antibodies to detect the membranous-cytoplasmic (1:100; mouse clone 2-18C9, DakoCytomation-EGFRPharmDx, Glostrup, Denmark) and the nuclear (1:100; Rabbit Polyclonal sc03, Santa Cruz Biotechnology Inc., Santa Cruz, CA, USA) localizations. For antigen retrieval, a citrate buffer of pH 9.0 was used. Endogenous peroxidase was cleared with 1% hydrogen peroxide, and positive reactivity of primary antibodies was performed by the HRP polymer and DAB as the chromogen substrate (Dako, Glostrup, Denmark).

EGFR and SGLT1 staining were scored semiquantitatively based on their staining intensity (0, negative; 1+, weak; 2+, moderate; 3+, strong) and percentage of stained cells (0–100). Membranous-cytoplasmic EGFR was positive when ≥1% of neoplastic cells showed positivity. The intensity of immunostaining was multiplied by the percentage of cell positive staining to design a TS between 0 and 300 for each tumor. A score ranging from 0 to 100 was considered weakly positive, from 101 to 200 moderately positive, and from 201 to 300 strongly positive [9].

### 4.3. Statistical Analysis

Statistical differences for qualitative variables were evaluated using Chi2 or Fisher’s Exact Test, when appropriate. Spearman’s correlations between membranous-cytoplasmic EGFR, nuclear EGFR, and SGLT1 expression levels were computed. Logistic regression analysis was performed to evaluate the association between progression of disease at 5 years of follow-up, clinico-pathological features and molecular variables.

Kaplan–Meier curve and Log-Rank test were performed to describe survival according to membranous-cytoplasmic EGFR, TS-EGFR, nuclear EGFR, and SGLT1 immunostaining. The statistical significance was set up at <0.05. Statistical analysis was carried out using STATA^®^16 (StataCorp, College Station, TX, USA).

## 5. Conclusions

The results of this study emphasize the roles of subcellular localization of EGFR and their correlation with clinico-pathological features of CCRCC. Membranous-cytoplasmic EGFR represents a negative prognostic factor. SGLT1 may influence the risk associated with EGFR kinase-independent function, defining a subgroup of CCRCC patients with good prognosis. The correlation between membranous-cytoplasmic and nuclear EGFR expression, and the favorable survival outcome in CCRCC patients with nuclear EGFR overexpression, could be an added value in selecting CCRCC patients with different outcomes. Membranous-cytoplasmic localization of EGFR could identify a subgroup of CCRCC patients with poor prognosis but who are potentially responsive to anti-EGFR therapies.

## Figures and Tables

**Table 1:** Clinico-pathological features of 120 patients with clear cell renal cell carcinoma (CCRCC).

Variables		n (%)
CCRCC	Italy	63 (52.5)
	Germany	57 (47.5)
Males		71 (59.2)
Mean (SD) age		62.0 (10.9)
Age ≥ 50 years		103 (85.8)
Median (IQR) size		6 (3.5–8.0)
Size > 5		68 (56.7)
Nuclear grading according to Fuhrman	1	13 (10.8)
	2	56 (46.7)
	3	42 (35.0)
	4	9 (7.5)
Pathologic tumor classification	pT1	58 (48.3)
	pT2	17 (14.2)
	pT3	45 (37.5)
Regional lymph nodes involvment	pNx	67 (55.8)
	pN0	45 (37.5)
	pN1	5 (4.2)
	pN2	3 (2.5)
Distant metastasis	M0	87 (72.5)
	M1	33 (27.5)
pTNM Stage	I	13 (16.1)
	II	6 (7.4)
	III	28 (34.6)
	IV	34 (42.0)
Coagulative tumor necrosis	Present	51 (44.0)
	Absent	65 (56.0)
Median (IQR) SSIGN		5 (2–7)
SSIGN (1)	0–2	39 (32.5)
	3–4	16 (13.39)
	5–6	29 (24.2)
	7–9	16 (13.3)
	≥10	20 (16.7)
SSIGN (2)	0–9	100 (83.3)
	≥10	20 (16.7)
Mean (SD) EGFR TS		177.5 (78.1)
EGFR TS	0–100	20/117 (17.1)
	101–200	50/117 (42.7)
	>201	47/117 (40.2)
SGLT1	Present	102 (85.0)
	Absent	18 (15.0)
EGFR nuclear	Present	11/57 (19.3)
	Absent	46/57 (80.7)
EGFR membranous	Present	111 (92.5)
	Absent	9 (7.5)
Follow-up 5 years	AWD	32/119 (26.9)
	DOD	8/119 (6.7)
	NED	79/119 (66.4)

n: number of patients; SD: standard deviation; pTNM: Pathological tumor-node-metastasis; IQR: interquartile range, Stage, Size, Grade, and Necrosis (SSIGN) score; AWD: alive with disease; DOD: disease-related deaths; NED: no evidence of disease.

**Table 2:** Correlation between EGFR total score and clinico-pathological features of patients with CCRCC.

Variables	rho	*p*-Value
Males	0.07	0.470
Age, years	−0.15	0.120
Tumor size	0.15	0.120
Nuclear grading according to Fuhrman	0.35	**0.0001**
Pathological Tumor classification	0.21	**0.020**
Regional lymph nodes involvement	0.08	0.590
Distant metastasis	0.12	0.220
pTNM stage	0.12	0.310
Coagulative tumor necrosis	−0.03	0.720
SSIGN score	0.25	**0.007**
Presence of SGLT1	−0.06	0.500
Presence of EGFR nuclear	−0.31	**0.020**

The *p* values are bold where they are less than or equal to the significance level of 0.05.

**Table 3:** Correlation between EGFR nuclear expression and clinico-pathological features of patients with CCRCC.

Variables	SGLT1					
	Absent n (%)			Present n (%)		*p*-Value
Males	7/29 (24.1)			4/28 (14.3)		0.500
Age ≥ 50 years	0/3 (0.0)			11/54 (20.4)		1.000
Tumor size > 5	8/32 (25.0)			3/25 (12.0)		0.320
SSIGN score ≥ 10	11/54 (20.4)			0/3 (0.0)		1.000
Coagulative tumor necrosis	2/23 (8.7)			9/30 (30.0)		0.090
**Regional lymph nodes involvement**	**pN0**			**pN1**		***p*-value**
Presence of EGFR nuclear, n (%)	3/12 (25.0)			0/1 (0.0)		1.000
**Distant metastasis**	**M0**			**M1** 27/33 (81.8)		***p*-value**
Presence of EGFR nuclear, n (%)	11/52 (21.2)			0/5 (0.0)		0.570
**Nuclear grading according to Fuhrman**	**G1**	**G2**			**G3–G4**	***p*-value**
Presence of EGFR nuclear, n (%)	3/12 (25.0)	6/29 (20.7)			2/16 (12.5)	0.750
**Pathological Tumor classification**	**pT1**	**pT2**			**pT3**	***p*-value**
Presence of EGFR nuclear, n (%)	8/35 (22.9)	1/6 (16.7)			2/16 (12.5)	0.780
**pTNM stage**	**1**	**2**	**3**		**4**	***p*-value**
Presence of EGFR nuclear, n (%)	1/3 (33.3)	1/1 (100.0)	2/14 (14.3)		0/5 (0.0)	0.160

**Table 4:** Correlation between SGLT1 expression and clinico-pathological features of patients with CCRCC.

Variables	SGLT1							
	Absent n (%)	Present n (%)						*p*-Value
Males	44/49 (89.8)	58/71 (81.7)						0.220
Age ≥ 50 years	14/17 (82.4)	88/103 (85.4)						0.720
Tumor size > 5	46/52 (88.5)	56/68 (82.4)						0.350
SSIGN score ≥ 10	84/100 (84.0)	18/20 (90.0)						0.730
Coagulative tumor necrosis	53/65 (81.5)	45/51 (88.2)						0.320
**Regional lymph nodes involvement**	**pN0**	**pN1**				**pN2**		***p*-value**
Presence of SGLT1, n (%)	40/45 (88.9)	5/5 (100.0)				3/3 (100.0)		1.000
**Distant metastasis**	**M0**			**M1**				***p*-value**
Presence of SGLT1, n (%)	75/87 (86.29)			27/33 (81.8)				0.550
**Nuclear grading according to Fuhrman**	**G1**	**G2**			**G3–G4**			***p*-value**
Presence of SGLT1, n (%)	11/13 (84.6)	47/56 (83.9)			44/51 (86.3)			0.940
**Pathological Tumor classification**	**pT1**	**pT2**			**pT3**			***p*-value**
Presence of SGLT1, n (%)	54/58 (93.1)	13/17 (76.5)			35/45 (77.8)			0.040
**pTNM stage**	**1**	**2**	**3**				**4**	***p*-value**
Presence of SGLT1, n (%)	12/13 (92.3)	6/6 (100.0)	20/28 (71.4)				28/34 (82.4)	0.310

The *p* values are bold where they are less than or equal to the significance level of 0.05.

**Table 5:** Logistic regression to assess the relationship between AWD or DOD and clinicopathological features in CCRCC patients.

Variables	OR (95% CI)	*p*-Value	OR (95% CI)	*p*-Value
Males	1.5 (0.7–3.2)	0.33		
Age, years	1.0 (0.9–1.0)	0.37	1.0 (0.9–1.1)	0.85
Tumor size	1.3 (1.1–1.4)	**0.001**	1.0 (0.8–1.3)	0.78
Nuclear grading according to Fuhrman	3.5 (1.9–6.4)	**<0.0001**	0.4 (0.1–1.2)	0.11
Pathological Tumor classification	2.0 (1.3–3.0)	**0.002**	0.2 (0.1–0.6)	**0.003**
Regional lymph nodes involvement	5.8 (0.8–41.1)	0.08		
Distant metastasis	-	-		
pTNM stage	-	-		
Coagulative tumor necrosis	1.5 (0.7–3.2)	0.30		
SSIGN score	1.8 (1.5–2.2)	**<0.0001**	2.7 (1.8–4.0)	**<0.0001**
EGFR total score	1.0 (1.0–1.0)	0.07		
Presence of EGFR membranous	1.8 (0.4–9.3)	0.46		
Presence of EGFR nuclear	-	-		
Presence of SGLT1	0.6 (0.2–1.6)	0.30		

OR: odds ratio; CI: confidence interval. The *p* values are bold where they are less than or equal to the significance level of 0.05.

**Table 6:** Correlation between membranous EGFR, nuclear EGFR and SGLT1 expression levels combination and CCRCC patients with NED, AWD or DOD.

**EGFRm/EGFRn**	**NED**	**AWD/DOD**	***p*-Value**
+/+, n (%)	9 (20.0)	0 (0.0)	0.09
+/−, n (%)	33 (73.3)	11 (91.7)	0.26
−/+, n (%)	2 (4.4)	0 (0.0)	1.00
−/−, n (%)	1 (2.2)	1 (8.3)	0.38
**EGFRm/SGLT1**	**NED**	**AWD/DOD**	***p*-Value**
+/+, n (%)	62 (78.5)	30 (75.0)	0.67
+/−, n (%)	10 (12.7)	8 (20.0)	0.29
−/+, n (%)	7 (8.9)	2 (5.0)	0.72
−/−, n (%)	-	-	-
**EGFRn/SGLT1**	**NED**	**AWD/DOD**	***p*-Value**
+/+, n (%)	10 (22.2)	0 (0.0)	0.10
+/−, n (%)	1 (2.2)	0 (0.0)	1.00
−/+, n (%)	32 (71.1)	9 (75.0)	1.00
−/−, n (%)	2 (4.4)	3 (25.0)	0.06

## Data Availability

Not applicable.

## Abbreviations

CCRCC	Clear cell renal cell carcinoma
RCC	Renal cell carcinoma
EGFRvIII	EGFR-variant III
NED	No evidence of disease
AWD	Alive with disease
DOD	Died of disease
TS	Total score
IHC	Immunohistochemistry
FFPE	Formalin-fixed, paraffin embedded
pT	Pathologic stage
SSIGN	Stage, size, grade, and necrosis
TKIs	Tyrosine kinase inhibitors
SD	Standard deviation
pTNM	Pathological tumor-node-metastasis
IQR	Interquartile range
OR	Odds ratio
CI	Confidence interval
PPP	Pentose phosphate pathway
